# Purification, HR-LC-ESI-MS-MS Identification, and Peptide Prediction of Bacteriocin-Like Inhibitory Substances Produced by *Streptomyces* sp. Isolated from *Chanos chanos*

**DOI:** 10.1155/2022/8672643

**Published:** 2022-08-02

**Authors:** Muhammad Alfid Kurnianto, Hanifah Nuryani Lioe, Ekowati Chasanah, Harsi Dewantari Kusumaningrum

**Affiliations:** ^1^Food Science Study Program, Graduate School, IPB University, Bogor, Indonesia; ^2^Department of Food Science and Technology, Faculty of Agricultural Engineering and Technology, IPB University, Bogor, Indonesia; ^3^Research and Development Center for Marine and Fisheries Product Processing and Biotechnology, Ministry of Marine and Fisheries, Jakarta, Indonesia

## Abstract

Consumption of fresh and minimally processed food is closely related to foodborne diseases. To minimize the adverse effects, bacteriocin-like inhibitory substance (BLIS) as a natural preservative can be used. One of the bacteriocins with promising activity was produced by *Streptomyces* sp. Using gel filtration chromatography, the bacteriocin purification process succeeded in obtaining semi-purified fractions with broad-spectrum inhibitory activity to foodborne pathogen bacteria. These fractions are also stable up to 100 °C and pH 2.0–7.0. High-Resolution Liquid Chromatography Electrospray Ionization-Tandem Mass Spectrometry analysis followed by orthogonal projection to latent structure showed that each fraction had eight peaks with the highest positive correlation to BLIS-specific activity. Peptide identification based on MS spectrum found 597 predictive peptides, of which 42 predictive peptides with antimicrobial peptide characteristics and the highest iAMPpred antimicrobial peptide probability (>0.5) were selected. The selected predictive peptides have molecular mass of 247.13-615.37 Da and consist of at least 20% hydrophobic amino acids with a hydrophobicity value of 14.72 Kcal mol^−1^. The results of this study indicate the effectiveness of BLIS purification by gel filtration chromatography and the promising potential of semi-purified BLIS as a natural preservative. Besides, the active peptides in semi-purified BLIS can also be identified quickly so that the isolation process to obtain purified-BLIS can be carried out more efficiently.

## 1. Introduction

Trends in healthy lifestyles lead to increased consumption of fresh food. However, since it is consumed without adequate processing, the potential for health problems due to infection with pathogenic microbes increases [1]. From 2010 to 2017, the Center for Disease Control and Prevention (CDC) confirmed a total of 1797 cases of food-borne outbreaks in the United States, of which 228 (12.7%) were fresh food-related [2]. Some pathogenic bacteria such as *Listeria monocytogenes*, *Escherichia coli*, *Staphylococcus* spp., and *Salmonella enterica* are commonly associated with food-borne disease outbreaks associated with fresh produce [1]. Various strategies were implemented to overcome these problems, such as physical treatment, chemical preservatives, and bio-preservatives. Bio-preservatives such as bacteriocins are among the best choices because they offer promising activity, stability, and safety [3].

Bacteriocins-like inhibitory substances (BLIS) are proteinaceous compounds synthesized ribosomally and secreted extracellularly by bacteria to inhibit other closely related bacteria [4]. The biochemical characteristics divide bacteriocins into three main classes: class I is the lantibiotic family, class II is small unmodified peptides that are heat resistant, and class III is large heat-labile proteins [5]. In general, bacteriocins have a narrow antibacterial spectrum and act through their interaction with “bacteriocin receptor” proteins on the cell membrane of target bacteria, causing membrane leakage and cell death [6, 7]. Most of the bacteriocins that have been found are produced by lactic acid bacteria (LAB) such as *Lactococcus*, *Streptococcus*, *Pediococcus*, and *Lactobacillus* [8]. Studies on the activity, stability, and safety of bacteriocins produced by LAB have been massively carried out. However, only two bacteriocins have been approved as food additives, pediocin and nisin [9]. Therefore, the exploration and identification of new bacteriocins are the focus of researchers' attention.

*Streptomyces* is one of the potential genera outside the LAB group, which is still little considered for its ability to synthesize bacteriocins. *Streptomyces* is filamentous Gram-positive bacteria. These bacteria have been known to produce promising bioactive compounds [10]. The study of Hernandez-Saldana et al. [11] showed that *S. griseus* and *S. nigrescens* produced stable bacteriocins at high temperatures and could inhibit food-borne pathogenic bacteria. Other studies have also shown that bacteriocins produced by *Streptomyces* have a broad spectrum of inhibition and can inhibit resistant pathogenic bacteria [12–14]. Despite its promising potential, few studies have reported its bioactivity, structural characteristics, synthesis processes, and potential applications.

In this study, purification of BLIS was carried out using gel filtration chromatography to obtain a semi-purified fraction. The semi-purified fraction was analyzed for antibacterial activity and protein concentration to the specific activity, and characterized for stability and toxicity (LC_50_). The semi-purified fraction was also predicted for its constituent peptides by High-Resolution Liquid Chromatography Electrospray Ionization-Tandem Mass Spectrometry (HR-LC-ESI-MS-MS) analysis followed by orthogonal projection to latent structure. Predicted peptides obtained were also analyzed for physicochemical properties *in silico*.

## 2. Materials and Methods

### 2.1. Preparation of Test Bacteria

The test bacteria are as follows: *Escherichia coli* ATCC 25922, *Staphylococcus aureus* ATCC 25922, Salmonella Typhimurium ATCC 14028, and *Listeria monocytogenes* were cultured in tryptic soy broth for 24 hours at 37 °C. Cultures of test bacteria that had grown were taken by loop, inoculated onto slanted agar containing tryptic soy agar medium (TSA; Oxoid, UK), and incubated for 24 hours at 37 °C [15].

### 2.2. Fractionation BLIS by Gel Filtration Chromatography

BLIS produced by six *Streptomyces* isolated from the gut of *Chanos chanos*: *S. variabilis* SCA5 (S5), *S. variabilis* SCA11 (S11), *S. variabilis* AIA10 (A10), *S. labedae* SCA8 (S8), *S. globisporus* AIA12 (A12) and *S. misionensis* AIA17 (A17) [15–17] with the method and culture conditions according to Kurnianto et al. [18]. BLIS in freeze-dried form dissolved in distilled water. The active fraction of BLIS was injected into the AKTA purifier system (GE Healthcare Sweden) with Sephadex G-25 matrix packed in a 1.6 × 70 cm column and eluted with phosphate buffer at a constant flow rate of 0.5 mL min^−1^. Sephadex G-25 has a fractionation range for proteins of molecular weights 1 to 5 kDa. The use of Sephadex G-25 is based on the molecular weight of the target compound and the fractionation range of the matrix. The absorbance was measured at 280 nm (for each 5 mL of eluates) in the AKTA purifier system, and the BLIS-GF chromatograms were analyzed with UNICORN software [19, 20]. The semi-purified BLIS-gel filtration fractions (BLIS-GF) was collected and grouped according to the peaks that appeared on the chromatogram and then freeze-dried. Each BLIS-GF volume was adjusted according to the initial volume to be tested for antibacterial activity and protein concentration.

### 2.3. Determination of Protein Concentration

The BLIS-GF (160 *μ*L) was reacted with Bradford's solution (40 *μ*L) in a 96-well microtiter plate and incubated at 37 °C for 10 min. The mixed solutions were analyzed using an ELISA reader (iMark™ Microplate Absorbance Reader 1681135, Bio-rad, US) at 595 nm. The same treatment was carried out on standard solutions of BSA (bovine serum albumin) and distilled water as blanks. Protein concentration was calculated as *μ*g mL^−1^. The protein concentration was then used for the calculation of BLIS specific activity [21].

### 2.4. Determination of Antibacterial Activity

The agar well diffusion method was used to determine the antibacterial activity of the BLIS-GF. Mueller Hinton agar media inoculated with the test bacteria (1 × 10^6^ CFU mL^−1^) was poured onto the disc and allowed to solidify, and wells were made (6 mm in diameter). 100 *μ*L of BLIS-GF was added to wells and incubated at 37 °C overnight. The diameter of the inhibition zone (mm) was measured. Based on the inhibition zone and protein concentration, the value of BLIS activity and the specific activity of BLIS can be determined [21]. BLIS activity is calculated using Eq. (1):

(1) $$\begin{array}{c} BLIS\;activity\;\left(A\right)=L_{z}–L_{s}/V, \end{array}$$

where *L*_z_ is the clear zone area, *L*_s_ is the well area, and *V* is the sample volume. The BLIS specific activity (ratio of total BLIS activity to total protein concentration) is calculated using Eq. (2):

(2) $$\begin{array}{c} Specific\;activity\;\left(B\right)=A\times V/T_{p}\times V, \end{array}$$

where *A* is BLIS activity, *T*_p_ is protein concentration, and *V* is sample volume.

### 2.5. Stability Analysis against High Temperature and Wide Range of pH

The pH stability was analyzed by adjusting the pH of the BLIS to pH 2.0–10.0, and incubating it for 2 hours at room temperature. Before being tested for its antibacterial activity using the agar well diffusion method, the pH was adjusted back to pH 7.0 [22]. In the heat stability analysis, BLIS were incubated at 121 °C for 15 min and at 60, 80, and 100 °C for 30 min. After being treated, the samples waited until they reached room temperature before being tested for their antibacterial activity using the agar well diffusion method [23].

### 2.6. Toxicity Analysis Using Brine-Shrimp Lethality Assay

A total of 10 *A. salina* larvae in test tubes were added with BLIS at a concentration of 1 mg mL^−1^, 500 *μ*g mL^−1^, 100 *μ*g mL^−1^, and 10 *μ*g mL^−1^ and incubated for 24 hours. After the incubation period was completed, a number of live and dead *A. salina* larvae were counted. The LC_50_ value was determined by Probit analysis at a 95% confidence interval using the SPSS program [23].

### 2.7. Peptide Identification by HR-LC-ESI-MS-MS

The BLIS-GF was dissolved in 1 mL of H_2_O (LC-MS grade), centrifuged for 1 min, and injected into NanoLC Ultimate 3000 tandem Q Exactive Plus Orbitrap HRMS (High Resolution Mass Spectrometry) with a Thermo PepMap RSLC C18 capillary column (75 *μ*m × 15 cm, 3 *μ*m, 100 Å) and trap column Thermo Scientific™ 164649 (30 *μ*m, 5 mm). The sample was eluted with H_2_O (LC-MS grade), 0.1% formic acid (A), and acetonitrile, 0.1% formic acid (B), at a flow rate of 300 nl min^−1^. The elution gradient used was 2%-35% B for 30 min, 30-90% B for 15 min, 90% B for 15 min, and 5% B for 30 min. The mass range used is 200-2000 m z^−1^. The High-Resolution Liquid Chromatography Electrospray Ionization-Tandem Mass Spectrometry (HR-LC-ESI-MS-MS) chromatogram data in the form of peak height was manually annotated using OriginPro 2019 and XCalibur software and written into Microsoft Excel. This data is used as matrix X. The antibacterial activity data (BLIS-specific activity) is relative to the highest antibacterial activity to get the percentage of antibacterial activity. This data is used as matrix Y. The data were analyzed by multivariate analysis *orthogonal projection to latent structure* (OPLS) [24]. OPLS analysis was conducted using SIMCA software (ver. 14.1; Umetrics, Sweden).

### 2.8. Prediction of Peptide Sequence, Physicochemical Characteristics, and Bioactivity Prediction

The peptide sequences were predicted based on the mass to charge ratio (m/z) of the MS spectrum of each peak on the HR-LC-ESI-MS-MS chromatogram [25]. The predicted peptide sequences were identified for their presence in the parent protein using peptide search tools from the Uniprot database, and the accession code for the parent protein was obtained. Based on the accession code, the molecular weight was confirmed on Findpept with a mass tolerance parameter of +0.12 Da, monoisotopic mass, and interpreted as a positive mode [M + H]+. The physicochemical properties of the predicted peptides, including sequence length, molecular weight, net charge, and hydrophobicity, were determined using Pepdraw (https://www.tulane.edu/~biochem/WW/PepDraw/) and Findpept (https://web.expasy.org/findpept/) following the identification conducted by Tamam et al. [25]. The peptide was also predicted to be an antibacterial peptide using iAMPpred following the prediction conducted by Kusumaningtyas and Dt [26].

### 2.9. Data Analysis

One-way ANOVA at a significance level of 0.05 was performed with IBM SPSS Statistics 24 software to determine potential BLIS. The orthogonal partial least square (OPLS) prediction model with Pareto scaling was carried out to identify the peaks that were most correlated with antibacterial activity (*E. coli*, *S. aureus*, *L. monocytogenes*, and *S.* Typhimurium). OPLS analysis was performed with SIMCA software (ver. 14.1; Umetrics, Sweden).

## 3. Results and Discussion

### 3.1. Antibacterial Activity of BLIS-Gel Filtration Fraction

**Figure 1:**
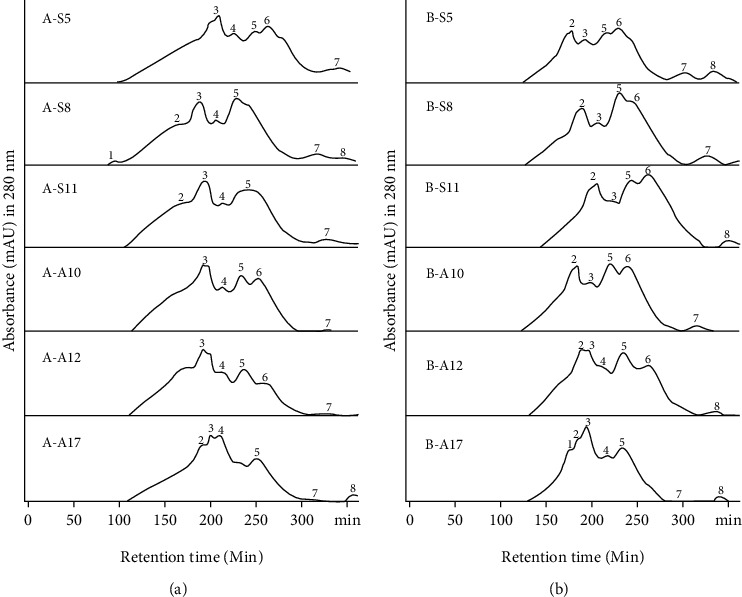
Fractionation of BLIS with gel filtration chromatography (Sephadex G-25); (a) BLIS-gel filtration fraction with MW 3–10 kDa, (b) BLIS-gel filtration fraction with MW <3 kDa; S5, S8, S11, A10, A12, and A17 are BLIS-producing isolates.

In a previous study, BLIS was fractionated using ultrafiltration membranes of 3 and 10 kDa. The initial fractionation process separates BLIS into fractions based on their molecular weight (MW), namely, fractions with MW <3 kDa, fractions with MW 3-10 kDa, and fractions with MW >10 kDa. Antibacterial activity and protein analysis showed that BLIS with MW <3 and 3-10 kDa were the most potent fractions because they had the highest BLIS-specific activity. Potential fractions also showed their sensitivity to proteolytic enzymes (proteinase-K, trypsin, and pepsin) [18]. The fraction was further fractionated by gel filtration chromatography to obtain semi-purified BLIS. This fractionation separated BLIS based on molecular mass, in which large molecules will elude faster [27, 28]. Further fractionation divided each BLIS-UF into eight BLIS-gel filtration (BLIS-GF) fractions based on the peak at 280 nm (Figure 1). The protein and antibacterial activity analysis showed the BLIS-specific activity (AU *μ*g^−1^), in which most of the highest activity was found in the eluted fraction at retention times between 90.0 to 220.0 min. Fourteen BLIS-GF fractions with potential and broad-spectrum BLIS-specific activity were selected (Table 1). The BLIS-GF B-A12-2 fraction with MW <3 kDa produced by *S. misionensis* A12 had the highest BLIS-specific activity against *E. coli*, *S. aureus*, *L. monocytogenes*, and *S*. Typhimurium of 1090.1, 1066, 659.7, and 579.6 AU *μ*g^−1^ (Table 1). These results are similar to those of Claesen and Bibb [12] and Hernandez-Saldana et al. [11]. They showed that BLIS produced by *S. griseus* and *S. nigrescences* had a low molecular mass (1.8 to 3 kDa) and had a broad inhibitory spectrum against *B. cereus*, *V. parahaemolyticus*, and *L. monocytogenes*.

Besides demonstrating the ability of BLIS, BLIS-specific activity can also show the purification process's effectiveness. In general, the BLIS-specific activity increased up to 21.4-fold after purification using ultrafiltration membranes and gel filtration chromatography (Table 1). The significant increase (*p* < 0.05) in activity indicates that the steps taken to purify BLIS were quite effective. Several studies with different purification methods also showed an increase in activity. Elayaraja et al. [29] stated that the purification of bacteriocin AU06 with ammonium sulphate precipitation method, DEAE-cellulose, and gel filtration chromatography Sephadex G75 were able to increase activity up to 4.74 times. Meanwhile, purification of bacteriocin M1-UVs300 with ATPS and Sephadex G50 method resulted in a 20.4-fold increase in activity [30].

### 3.2. BLIS-Gel Filtration Fraction Stability to High Temperature and Wide Range of pH

Stability analyses at high temperature showed that 9 of the 14 selected BLIS-GF fractions maintained more than 50% of their antibacterial activity up to 100 °C for 30 min. In fact, in the BLIS-GF A-S5-3 fraction produced by *S. variabilis* SCA5 and B-S8-2 produced by *S. labedae* SCA8, the antibacterial activity persisted up to 121 °C for 15 min (Table 2). In pH stability analysis, most BLIS-GF fractions maintained at least 50% antibacterial activity at pH 2.0 to 7.0 (Table 2). The stability of BLIS-GF in this study was consistent with the study of Hernandez-Saldana et al. [11], in which BLIS produced by *S. nigrescens* was able to maintain almost half its activity at 120 °C and in the pH range of 3.0–10.0. Stability to high temperatures and pH is thought to be caused by several factors such as secondary structure, thermostable amino acid content, and changes in charge due to the influence of isoelectric point [31, 32]. Considering that most food products have a neutral to acidic pH and are processed using a heat process [33], this study showed the potential for use in food preservation processes.

### 3.3. Lethal Concentration (LC_50_) of BLIS-Gel Filtration Fractions

Brine shrimp lethality assay is a toxicity test method commonly used in the initial evaluation of the bioactivity of a substance [34]. The test results were expressed as LC_50_ (median lethal concentration), which indicates the concentration of a compound that can kill 50% of the test organisms. The test results showed that the LC_50_ of selected BLIS-GF fractions was between 93.9 and 707.5 *μ*g mL^−1^, which were in the category of high to low toxicity to *Artemia salina* (Table 2). Hamidi et al. [35] classified toxicity based on the LC_50_ value into four categories: high toxicity (LC_50_ 0–100 *μ*g mL^−1^), moderate toxicity (LC_50_ 100–500 *μ*g mL^−1^), low toxicity (LC_50_ 500–1000 *μ*g mL^−1^), and non-toxic (LC_50_>1000 *μ*g mL^−1^). Parra et al. [36] reported that the LC_50_ from the brine shrimp lethality assay and the LD_50_ from the acute oral toxicity test in animal models had a positive correlation, in which LC_50_ > 25 *μ*g mL^−1^ has an LD_50_ of 2500–8000 mg kg^−1^. Based on the study, selected BLIS-GF was in the category of low toxicity [37]. Therefore, BLIS in form semi-purified BLIS (BLIS-GF) had the potential to be developed as a natural food preservative.

### 3.4. HR-LC-ESI-MS-MS – Multivariate Analysis

This study uses multivariate orthogonal projection to latent structure (OPLS) analysis which correlates the High-Resolution Liquid Chromatography Electrospray Ionization-Tandem Mass Spectrometry (HR-LC-ESI-MS-MS) profile (variable *X*) with BLIS-specific activity (variable *Y*). In this analysis, the HR-LC-ESI-MS-MS profile (% relative abundance) was divided into predictive and orthogonal models. The predictive model correlated the variables *X* with *Y* associated, and the orthogonal model represented the variable *X* that was not associated with *Y* [38]. The values of R2Y and Q2 are used to determine the quality of the model. R2Y indicates the fitness level and the number of *Y* variables that can be explained by the model, while Q2 shows the prediction of the quality of the model [39]. OPLS analysis was interpreted using a score plot to show the separation between fractions, an S plot to show the peaks responsible for bioactivity, and a Y-related coefficient plot to study the correlation of variables *X* and *Y* [39].

**Figure 2:**
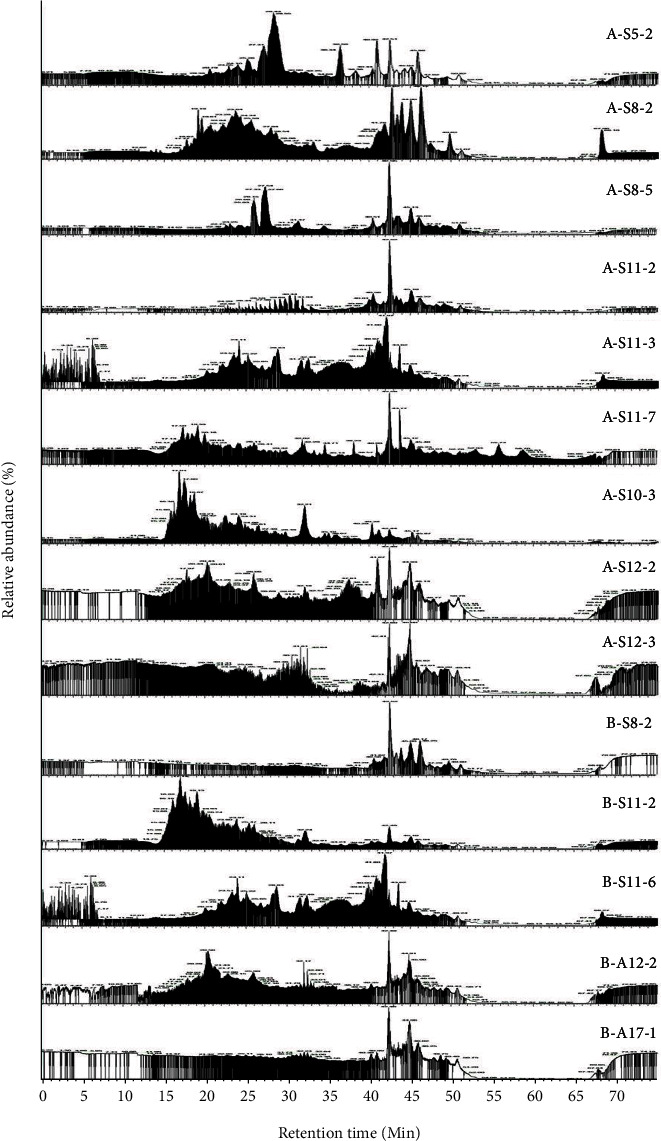
The HR-LC-ESI-MS-MS chromatogram of selected BLIS-GF fractions with potential BLIS-specific activity; (a) BLIS-gel filtration fraction with MW 3–10 kDa, (b) BLIS-Gel filtration fraction with MW <3 kDa; S5, S8, S11, A10, A12, and A17 are BLIS-producing isolates.

**Figure 3:**
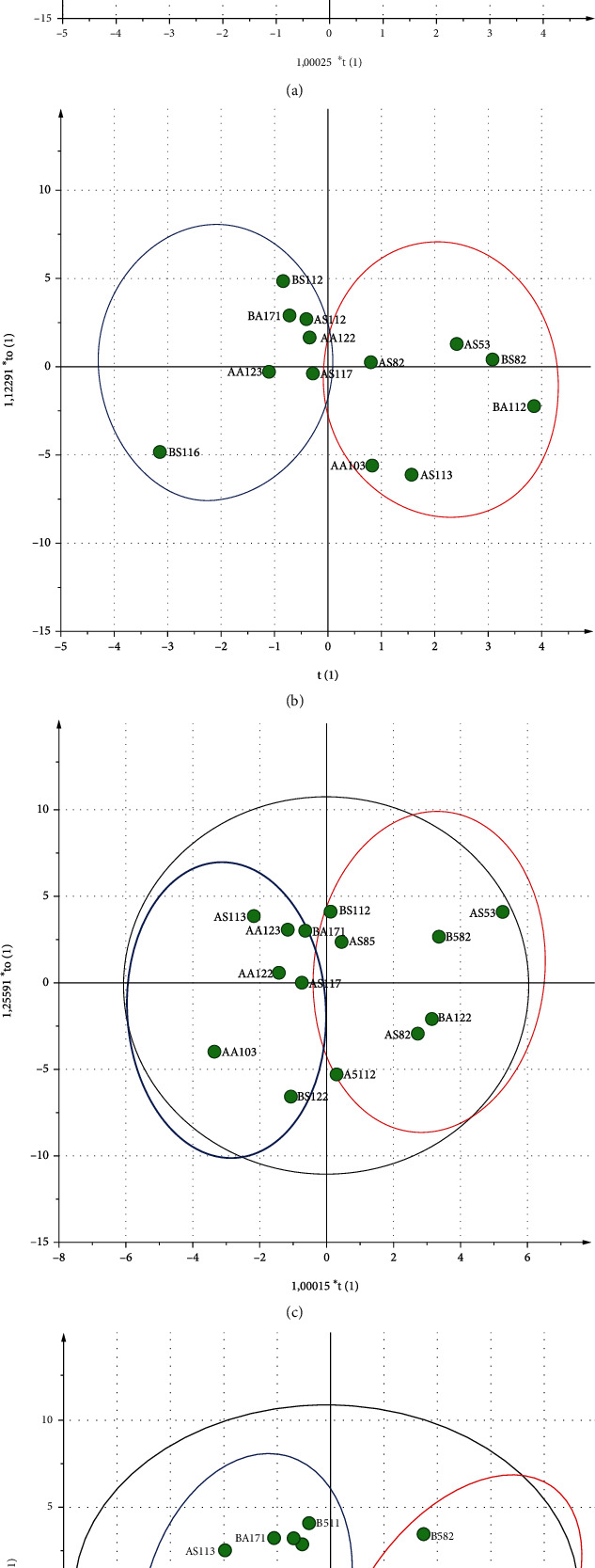
The score-plot output from OPLS analysis showed the distribution of selected BLIS-GF fractions based on BLIS-specific activity. Blue: BLIS-GF with high BLIS-specific activity; red: BLIS-GF with low BLIS-specific activity; *E. coli* (a), *S. aureus* (b), *L. monocytogenes*, and (c) *S.* Typhimurium (d).

**Figure 4:**
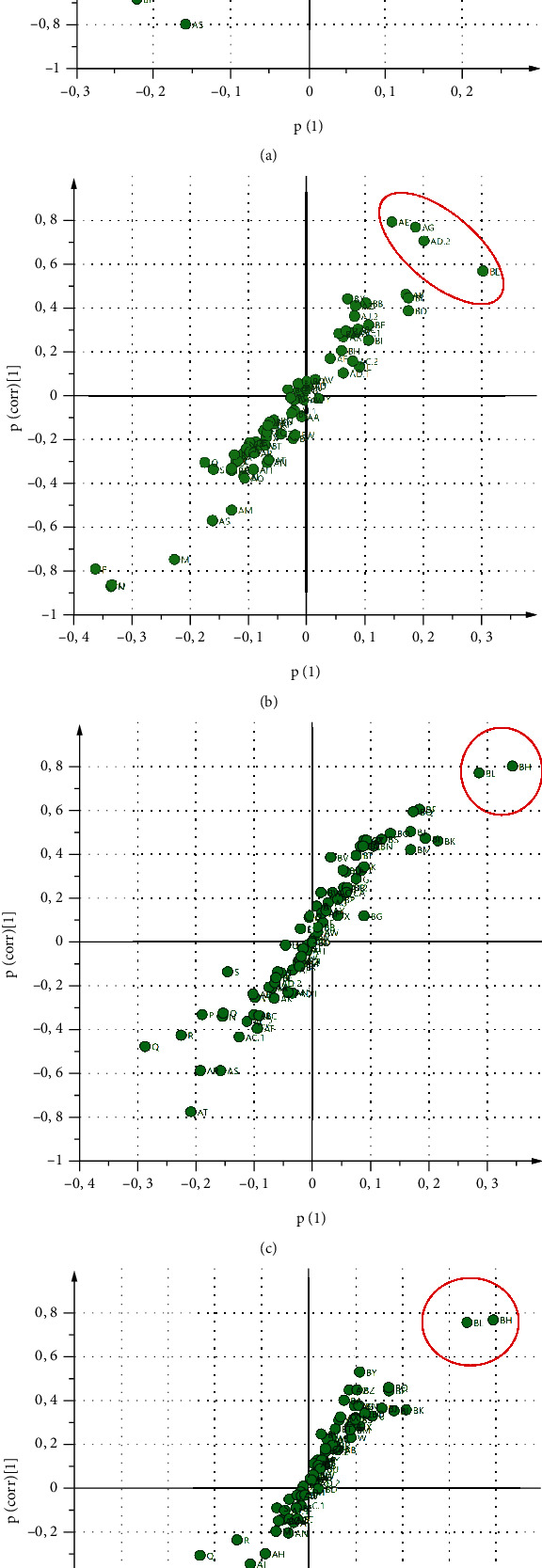
Correlation plot HR-LC-ESI-MS-MS chromatogram and BLIS-specific activity. The red circle indicates the peak with the strongest correlation; *E. coli* (a); *S. aureus* (b); *L. monocytogenes* (c); and *S.* Typhimurium (d).

Identifying the fractions by HR-LC-ESI-MS-MS resulted in a chromatogram output having 82 peaks with different relative abundances (Figure 2). OPLS analysis showed that R2Y and Q2 values of the model are in range of 0.901–0.989 and 0.526–0.604, respectively, which indicated the model's goodness. In addition, model validation using cross-validated-ANOVA (CV-ANOVA) showed that the model was significant (*p* < 0.05) [40]. Based on the score-plot, BLIS-GF fractions separate the active and less active fractions (Figure 3). The separation results indicate that the OPLS model is suitable for identifying the active fraction [24]. At the same time, the S-plot and Y-related coefficient plot identified the peaks responsible for the antibacterial activity of each test bacterium (Figure 4). The peaks were ab (22.80 min) and ad.1 (24.11 min) on antibacterial activity against *E. coli*; ad.2 (25.26 min), ae (25.59 min), ag (26.61 min), and b (40.99 min) on antibacterial activity against *S. aureus*; and bh (43.51) and bl (45.86 min) on antibacterial activity against *L. monocytogenes* and *S.* Typhimurium (Figure 4).

### 3.5. Peptide Sequence Prediction

The peptide sequence responsible for the antibacterial activity was identified based on the mass to charge ratio (m/z) in the MS spectrum of each peak on the HR-LC-ESI-MS-MS chromatogram. The identification results showed that 597 predictive peptides were obtained (data not shown). A total of 42 predictive peptides (each fraction consisting of three peptides responsible for *E. coli*, *S. aureus*, *L. monocytogenes*, and *S.* Typhimurium, respectively) with similar characteristics to antimicrobial peptides and having a high probability (>0.5) as antimicrobial peptides based on iAMPpred predictions were selected (Table 3). Most of the peptides found were dipeptides and tripeptides with low molecular mass ranging from 247.13 to 615.37 Da. Besides, the peptides also have 20% hydrophobic amino acids with the hydrophobicity of 5.60–14.72 Kcal mol^−1^ and a net charge of − 2 to +2. According to Tamam et al. [41], several peptide characterizing parameters correlate with antibacterial activity. These parameters are amino acid composition, net charge, molecular weight, and hydrophobicity to the isoelectric point. In general, antimicrobial peptides are cationic and hydrophobic. Cationic properties play a role in the interaction of peptides, and the cell membranes of target bacteria and hydrophobic properties play a role in forming pore structures that cause cell death [42, 43]. In negatively charged (anionic) antimicrobial peptides, a cationic salt bridge formation mechanism is thought to facilitate the interaction of anionic peptides with target bacteria [44]. Besides antimicrobial peptides, especially bacteriocins generally have molecular weights ranging from 3 to 10 kDa [45]. However, several recent studies have shown that there are bacteriocins with molecular weight <1.5 kDa, such as bacteriocin SLG10 with 1422 Da [46], plantaricin GZ1-27 with 975 Da [47], and bifidocin A with 1198.68 Da [48]. Antimicrobial properties are also determined from the ratio of hydrophobic and cationic amino acids. The higher the ratio of hydrophobicity and cationic amino acids, the ability will increase [49]. Besides forming pores, antibacterial peptides with low molecular mass can also penetrate bacterial cell membranes and attack intracellular components [50].

## 4. Conclusions

BLIS fractionation using gel filtration chromatography found 14 BLIS fractions had potential BLIS specific activity with the broad antibacterial spectrum, good stability at high temperature and pH, and low toxicity (LD_50_). HR-LC-ESI-MS-MS–multivariate analysis followed by identification of predictive peptide sequences detected 597 peptides, of which 42 peptides with antimicrobial peptide-like characteristics and having the highest iAMPpred-based antimicrobial peptide probability (>0.5) were selected. The selected peptides had a low molecular mass (247.13–615.37 Da), a net charge of − 2 to + 2, at least 20% hydrophobic amino acids, and a hydrophobicity of up to 14.72 Kcal mol^−1^. Overall, the results of this study indicate that HR-LC-ESI-MS-MS–multivariate analysis followed by peptide prediction based on MS spectrum allows rapid identification of peptides responsible for antibacterial activity. This allows the isolation process to obtain purified-BLIS to be carried out more efficiently.

## Figures and Tables

**Table 1:** BLIS activity, BLIS specific activity and purification level of selected BLIS-gel filtration fraction.

BLIS-GF fraction	Protein (*μ*g mL^−1^)	BLIS activity (AU mL^−1^)				BLIS-specific activity (AU *μ*g^−1^)				Purification fold			
		EC	SA	LM	ST	EC	SA	LM	ST	EC	SA	LM	ST
A-S5-3	15.7	9098.7	10029.8	13786	10283.5	580.6^d,A^	640.0^f,AB^	879.7^g,C^	656.2^i,B^	4.0	5.8	3.3	3.9
A-S8-2	11.9	8411.1	5536.5	7354	6312.4	709.1^e,D^	466.7^d,A^	619.9^f,C^	532.1^gh,B^	1.9	3.3	2.3	1.4
A-S8-5	14.7	7809.4	6067.7	5769.6	3541.6	530.5^d,B^	412.2^d,C^	392.0^e,B^	240.6^e,A^	1.4	2.9	1.5	0.6
A-S11-2	14.3	7178.7	8090.1	5219.7	4589.1	500.3^cd,B^	563.8^e,B^	363.8^e,A^	319.8^f,A^	1.6	5.2	1.4	1.1
A-S11-3	15.9	6280.2	5536.5	1759.7	1284.1	394.1^b,B^	347.4^c,B^	110.4^a,A^	80.6^b,A^	1.3	3.2	0.4	0.3
A-S11-7	17.9	7325.3	6067.7	5255.9	3226.2	409.4^b,C^	339.1^c,BC^	293.7^d,B^	180.3^cd,A^	1.3	3.1	1.1	1.1
A-A10-3	11.7	8676.0	5244.1	—	3147.5	744.7^e,C^	450.1^d,B^	—	270.2^ef,A^	2.6	7.3	—	2.0
A-A12-2	15.7	5998.3	3958.6	2900.1	4412.9	382.7^b,C^	252.6^b,AB^	185.1^b,A^	281.6^ef,B^	1.6	5.1	0.9	1.6
A-A12-3	23.4	8926.5	8070.1	5137.2	5478.8	381.5^b,C^	344.9^c,B^	219.6^bc,A^	234.2^de,A^	1.6	6.9	1.0	1.4
B-S8-2	14.4	7940.5	11206.2	9283.6	6890.3	551.3^d,B^	778.1^g,D^	644.5^f,C^	478.4^g,A^	1.5	5.6	2.4	1.3
B-S11-2	39.6	8940.0	11731.8	14721.4	10795.8	225.^5a,A^	295.9^bc,A^	371.3^e,B^	272.3^ef,A^	0.7	2.7	1.4	1.4
B-S11-6	14.6	6571.5	341.7	3373.0	290.0	451.3^bc,C^	23.4^a,A^	231.6^bc,B^	19.9^a,A^	1.5	0.2	0.9	0.9
B-A12-2	8.6	9355.1	9148.3	5661.7	4973.9	1090.1^f,B^	1066^h,B^	659.7^f,A^	579.6^h,A^	4.5	21.4	3.1	3.1
B-A17-1	17.6	9441.5	5148.7	4895.6	3027.3	537.2^d^	292.9^bc^	278.5^cd^	172.2^c^	2.4	2.4	1.8	1.8

The BLIS-gel filtration fraction that was not shown showed little or no antibacterial activity against the 4 tested bacteria. Description of BLIS-GF fraction code name: A/B (beginning of code): fraction with of 3-10 kDa (A) or <3 kDa (B) as a result of separation with an ultrafiltration membrane; S5/S8/S11/A10/A12/A17 (middle of code): BLIS-producing isolates; numbers 1-7 (end of code): fraction number assigned based on the results of BLIS separation by gel filtration; (EC) *E. coli*; (SA) *S. aureus*; (LM) *L. monocytogenes*; (ST) *S*. Typhimurium. The different small letters in the same rows showed a significant difference (*p* < 0.05) between the BLIS-GF samples. The different capital letters in the same column show a significant difference (*p* < 0.05) between test bacteria.

**Table 2:** Toxicity (LC_50_) analysis and stability analysis of selected BLIS-gel filtration fraction against high temperature and wide range of pH treatment.

BLIS-GF	Control (mm)	Inhibition zone (mm) after high-temperature treatment				Inhibition zone (mm) after wide-range pH treatment						LC_50_ (*μ*g mL^−1^)
		60 °C	80 °C	100 °C	121 °C	2	4	6	7	8	10	
A-S5-3	27.05 ± 0.13^E,*E*^	19.18 ± 0.18^a,C^	20.58 ± 0.11^e,D^	18.48 ± 0.04^h^^,B^	16.85 ± 0.07^f,A^	22.84 ± 0.24^c,*C*^	25.82 ± 0.84^e,D^	25.58 ± 0.27^hi,*D*^	26.01 ± 0.13^g,*D*^	20.75 ± 0.14^i,*B*^	15.49 ± 0.28^h^^,*A*^	128.0^c^
A-S8-2	27.81 ± 0.08^E,*D*^	26.72 ± 0.14^h,D^	21.86 ± 0.05^g,C^	20.38 ± 0.04^j,B^	12.43 ± 0.04^d,A^	22.57 ± 0.17^c,*B*^	22.08 ± 0.38^cd,B^	25.72 ± 0.07^i,*C*^	25.41 ± 0.21^g,*C*^	19.22 ± 0.35^h,*A*^	19.98 ± 0.57^i,*A*^	93.9^a^
A-S8-5	25.16 ± 0.50^D,*F*^	24.38 ± 0.11^f,D^	20.60 ± 0.16^e,C^	9.47 ± 0.10^c,B^	0^a,A^	25.10 ± 0.19^ef,*F*^	21.13 ± 0.69^ab,*E*^	15.90 ± 0.65^a,*D*^	8.49 ± 0.18^a,*C*^	3.40 ± 0.07^a,*B*^	0^a,*A*^	139.9^d^
A-S11-2	24.18 ± 0.18^E,*DE*^	22.58 ± 0.06^d,D^	17.07 ± 0.04^b,C^	6.93 ± 0.11^a,B^	0^a,A^	23.89 ± 0.62^cde,*D*^	24.41 ± 0.27^d,*DE*^	25.06 ± 0.23^ghi,*E*^	20.74 ± 0.11^e,*C*^	18.33 ± 0.26^g,*B*^	15.84 ± 0.65^h,*A*^	258.1^i^
A-S11-3	22.72 ± 0.30^E,*D*^	20.82 ± 0.16^b,D^	18.52 ± 0.04^c,C^	16.25 ± 0.21^f,B^	6.90 ± 0.03^b,A^	23.81 ± 0.94^c,*D*^	25.50 ± 0.37^e,*E*^	23.71 ± 0.05^e,*D*^	18.05 ± 0.49^d,*C*^	15.97 ± 0.11^f,*B*^	13.97 ± 0.59^g,*A*^	549.4^m^
A-S11-7	24.41 ± 0.29^D,*E*^	23.45 ± 0.02^e,D^	16.51 ± 0.08^a,C^	6.94 ± 0.16^a,B^	0^a,A^	18.11 ± 0.41^a,*A*^	21.07 ± 0.31^ab,*C*^	24.18 ± 0.52^ef,*E*^	24.48 ± 0.25^f,*E*^	21.90 ± 0.28^j,*D*^	19.27 ± 0.40^i,*B*^	193.2^h^
A-A10-3	26.44 ± 0.33^E,*E*^	24.10 ± 0.08^f,D^	20.06 ± 0.02^d,C^	8.84 ± 0.16^b,B^	0^a,A^	20.55 ± 0.45^b,*D*^	25.99 ± 0.40^e,*E*^	20.55 ± 0.45^d,*D*^	8.05 ± 0.41^a,*C*^	3.98 ± 0.21^a,*B*^	0^a*,A*^	266.9^j^
A-A12-2	22.24 ± 0.37^E,*F*^	20.92 ± 0.02^b,D^	18.50 ± 0.13^c,C^	15.83 ± 0.11^f,B^	8.32 ± 0.04^c,A^	23.19 ± 0.42^cd,*F*^	23.96 ± 0.58^d,*F*^	18.44 ± 0.08^c,*D*^	12.49 ± 0.44^b,*C*^	9.94 ± 0.30^c,*B*^	7.32 ± 0.24^b,*A*^	707.5^n^
A-A12-3	26.80 ± 0.28^D,*E*^	25.38 ± 0.05^g,D^	21.38 ± 0.01^f,C^	11.19 ± 0.02^d,B^	0^a,A^	26.59 ± 0.18^g,*E*^	26.05 ± 0.71^e,*E*^	18.34 ± 0.05^c,*D*^	12.74 ± 0.06^b,*C*^	10.59 ± 0.22^d,*B*^	8.51 ± 0.46^d,*A*^	177.5^g^
B-S8-2	23.36 ± 0.29^C,*D*^	25.15 ± 0.07^g,E^	23.74 ± 0.06^i,D^	20.44 ± 0.05^j,B^	18.09 ± 0.16^g,A^	22.85 ± 0.35^c,*D*^	26.41 ± 0.30^e,*F*^	24.44 ± 0.22^fg,*E*^	20.88 ± 0.47^e,*C*^	15.62 ± 0.24^f,*B*^	10.41 ± 0.04^e,*A*^	151.3^e^
B-S11-2	26.82 ± 0.04^D,*E*^	25.38 ± 0.15^g,D^	22.63 ± 0.24^h,C^	19.33 ± 0.08^i,B^	7.87 ± 0.67^c,A^	20.55 ± 0.07^b,*B*^	23.99 ± 0.25^d,*C*^	26.64 ± 0.23^j,*E*^	25.68 ± 0.19^g,*D*^	20.72 ± 0.30^i*,B*^	15.88 ± 0.42^h,*A*^	316.5^l^
B-S11-6	23.20 ± 0.28^D,*E*^	21.63 ± 0.20^c,C^	16.92 ± 0.36^b,B^	14.29 ± 0.15^e,B^	7.00 ± 0.46^b,A^	24.75 ± 1.48^def,*E*^	22.68 ± 0.14^c,*E*^	18.50 ± 0.23^c,*D*^	13.05 ± 0.06^b,*C*^	10.42 ± 0.19^cd,*B*^	7.56 ± 0.23^c,*A*^	265.7^j^
B-A12-2	27.41 ± 0.13^C,*G*^	27.06 ± 0.73^h,C^	24.71 ± 0.28^j,BC^	21.08 ± 0.76^k,B^	14.10 ± 0.26^e,A^	25.86 ± 0.09^fg,*E*^	26.41 ± 0.05^e,*F*^	17.57 ± 0.02^b,*D*^	13.00 ± 0.27^b,*C*^	6.52 ± 0.11^b,*B*^	0^a,*A*^	100.3^b^
B-A17-1	24.65 ± 0.21^D,*F*^	24.98 ± 0.28^g,D^	20.49 ± 0.17^e,C^	17.62 ± 0.17^g,B^	14.12 ± 0.44^e,A^	22.67 ± 0.08^c,*E*^	20.64 ± 0.22^a,*D*^	24.92 ± 0.37^gh,*F*^	17.05 ± 0.33^c,*C*^	12.66 ± 0.31^e,*B*^	8.22 ± 0.38^d,*A*^	176.4^f^

Description of BLIS-GF Fraction code name: A/B (beginning of code): fraction with of 3-10 kDa (A) or <3 kDa (B) as a result of separation with an ultrafiltration membrane; S5/S8/S11/A10/A12/A17 (middle of code): BLIS-producing isolates; numbers 1-7 (end of code): fraction number assigned based on the results of BLIS separation by gel filtration. Means value ± SD (standard deviation) from three replications. The different small letters in the same rows showed significant differences (*p* < 0.05) between the BLIS-GF samples; different capital letters in the same column showed significant differences (*p* < 0.05) between treatments (pH or temperature).

**Table 3:** Peptide sequence prediction, in *silico* analysis of physicochemical properties and prediction antimicrobial peptide candidate.

BLIS-GF	Test bacteria	Peptide's sequence	Net charge^1^	Hydrophobicity^1^ (kcal mol^−1^)	Molecular mass (Da)^2^	Antimicrobial peptide prediction^3^
AS53	EC	**F**E	− 1	9.82	295.13	0.64
	SA	S**L**K	+1	9.91	347.23	0.86
	LM and ST	D**PL**D	− 2	14.07	459.21	0.64
AS82	EC	S**WG**	0	7.42	349.151	0.72
	SA	N**F**H	+1	9.37	417.19	0.64
	LM and ST	E**L**H	− 1	12.61	398.20	0.72
AS85	EC	**W**NR	+1	8.47	475.24	0.66
	SA	G**VY**	0	7.88	338.17	0.64
	LM and ST	D**VV**	− 1	10.62	331.17	0.64
BS82	EC	**L**D	− 1	10.29	247.13	0.65
	SA	RK**LM**	+2	10.59	546.78	0.72
	LM and ST	**W**K	+1	8.61	333.19	0.66
AS112	EC	**PW**K**Y**	+1	8.04	593.31	0.68
	SA	DHT**AY**	− 1	13.91	606.25	0.55
	LM and ST	D**W**N	− 2	10.30	434.17	0.64
AS113	EC	G**W**	0	6.96	262.12	0.66
	SA	**F**H	+1	8.52	303.15	0.64
	LM and ST	**LP**	0	6.79	229.16	0.58
AS117	EC	D**P**Q	− 1	12.45	359.16	0.61
	SA	H**C**K	+1	13.01	387.18	0.69
	LM and ST	G**P**K	+1	11.99	301.19	0.69
BS112	EC	**MP**	0	7.37	247.11	0.64
	SA	N**P**K**G**	+1	12.84	415.23	0.55
	LM and ST	**IF**TE**V**	− 1	8.49	608.33	0.20
BS116	EC	**MF**HR	+1	9.66	590.29	0.64
	SA	E**M**	− 1	10.86	279.10	0.64
	LM and ST	S**CF**	+1	6.63	356.13	0.64
AA103	EC	**I**GNRR	+2	12.27	615.37	0.73
	SA	K**F**N	+1	9.84	408.22	0.59
	LM and ST	RK**F**	+2	10.80	450.28	0.75
AA122	EC	**M**K**LA**K	+2	12.08	590.37	0.55
	SA	S**MW**	0	5.60	423.17	0.62
	LM and ST	R**I**	+1	8.59	288.20	0.52
AA123	EC	S**PF**	0	6.79	350.17	0.58
	SA	D**Y**	− 1	10.83	297.11	0.43
	LM & ST	C**FP**	− 1	6.31	366.148	0.67
BA122	EC	R**M**	+1	9.04	306.16	0.54
	SA	E**M**N	− 1	11.71	393.14	0.40
	LM and ST	HND	− 1	14.72	385.15	0.39
BA171	EC	E**L**	− 1	10.28	261.14	0.50
	SA	**L**D**PL**	− 1	9.18	457.27	0.57
	LM and ST	H**VL**T**C**	+1	8.75	572.29	0.78

Description of BLIS-GF fraction code name: A/B (beginning of code): fraction with of 3-10 kDa (A) or <3 kDa (B) as a result of separation with an ultrafiltration membrane; S5/S8/S11/A10/A12/A17 (middle of code): BLIS-producing isolates; numbers 1-7 (end of code): fraction number assigned based on the results of BLIS separation by gel filtration (EC) *E. coli*; (SA) *S. aureus*; (LM) *L. monocytogenes*; (ST) *S. typhimurium.* Bold letters indicate hydrophobic amino acid residues; prediction of physicochemical properties based on ^1^PepDraw and ^2^Findpep; prediction of antimicrobial peptide candidate based on ^3^iAMPpep; net charge is the sum of positively (basic) and negatively (acidic) charge residues in neutral pH; molecular mass is the sum of monoisotopic masses of all amino acid residue in the peptide; hydrophobicity (Wimley-White scale) is the free energy associated with transitioning a peptide from an aqueous to hydrophobic environment. Antimicrobial peptide prediction shows possibility of peptide sequences that are predicted to be antimicrobial. A value >0.5 indicates a high probability as antimicrobial peptide.

## Data Availability

All datasets generated or analyzed during this study are available upon reasonable request from the corresponding author.
